# Thermally Introduced Bismuth Clustering in Ga(P,Bi) Layers under Group V Stabilised Conditions Investigated by Atomic Resolution *In Situ* (S)TEM

**DOI:** 10.1038/s41598-018-27286-4

**Published:** 2018-06-13

**Authors:** R. Straubinger, M. Widemann, J. Belz, L. Nattermann, A. Beyer, K. Volz

**Affiliations:** Faculty of Physics and Materials Science Center, Philipps-Universität Marburg, Hans-Meerwein-Straße 6, Marburg, Hessen 35032 Germany

## Abstract

We report the formation of Bi clusters in Ga(P_1-x_,Bi_x_) layers during an *in situ* (scanning) transmission electron microscopy ((S)TEM) annealing investigation. The non-destructive temperature regime in dependence on the tertiarybutylphosphine (TBP) pressure in the *in situ* cell was investigated to ensure that the results are not distorted by any destructive behaviour of the crystal during the thermal treatment. The following annealing series of the Ga(P_92.6_Bi_7.4_) and Ga(P_96.4_Bi_3.6_) layers reveals that the threshold temperature at which the Bi clustering takes place is 600 °C in the Ga(P_92.6_Bi_7.4_) layer. Further thermal treatments up to 750 °C show a relationship between the Bi fraction in the Ga(P_1-x_,Bi_x_) layer and the initial temperature at which the Bi clustering takes place. Finally, we investigate one Bi cluster at atomic resolution conditions. In these conditions, we found that the Bi cluster crystallized in a rhombohedral phase, aligning with its {101} planes parallel to the Ga(P,Bi) {202} planes.

## Introduction

Bi containing III/V semiconductor materials have seen growing interest due to their strong reduction of the band gap energy even with a small fraction of Bi^1–3^. Furthermore, a growing Bi content leads to a drastic increase of spin-orbit splitting^4^. For example in GaAs the band gap is reduced by about 90 meV per percent of Bi incorporation^2^. With a Bi fraction higher than 10% the spin-orbit splitting becomes even larger than the band gap^5,6^. Due to this modification Auger recombination processes can be suppressed, leading to an increased efficiency in photonic devices. Several bismide materials showed similar properties to those mentioned ones in Ga(As,Bi) promising new applications in optoelectronics^7–11^. Further theoretical calculations on the properties Ga(P,Bi), which could be a promising candidate for tuning the emission of optical sources as well as for the fabrication of laser devices emitting in the telecommunication wavelength, were reported^12^. Since GaP can be grown nearly lattice matched on Si^13^ the possibility to grow Ga(P,Bi) devices on Si exists^14^. The fabrication of materials with a high fraction of Bi is, however, challenging due to its highly metastable nature. Furthermore, the grown Bi containing materials have to have a very high quality in terms of crystal defects to be practical for optoelectronic applications. The investigation by Christian *et al*.^15^ demonstrated a Bi incorporation of 3.2% in GaP using a molecular beam epitaxy (MBE) setup. Later results on metal organic vapor phase epitaxy (MOVPE) grown Ga(P,Bi) samples by Nattermann *et al*.^14^ showed a Bi incorporation of up to 8%. In addition, *in situ* information with regards to Bi cluster formation during growth and post growth annealing is missing. Furthermore, the mechanisms involved are not yet understood^16,17^. Beside all successes, the optimization of growth conditions for functional III/V semiconductor materials is still very challenging as can be seen by the growing body of publications within this field^18–20^. In particular *in situ* information promises to deliver details about the growth mechanisms and can therefore help to improve the growth parameters. One of the advantages of *in situ* methods is that they enable live insights into dynamic processes. *In situ* (scanning) transmission electron microscopy ((S)TEM) has been proven to be an outstanding technique for these kind of observations^21,22^. The compatibility of modern *in situ* holders with most TEMs makes this technique feasible for a wide range of research. *In situ* TEM systems, such as the Atmosphere system^23^ developed and produced by Protochips (Morrisville, NC, USA), are enabling observations at gas pressures up to 1,000 hPa and temperatures of 1,000 °C. With these specifications the experimental growth conditions in terms of temperature and precursor pressure of MOVPE, which is commonly used to fabricate novel III/V semiconductor compounds, can be realized.

In this paper we use *in situ* (S)TEM to investigate the threshold temperature at which the local Bi clustering take place. Further we investigate the cluster formation process as a function of the Bi fraction of the Ga(P,Bi) layers under group V stabilized conditions. Furthermore, we give a detailed insight into the Bi cluster characteristics under atomic resolution conditions.

## Experimental Methods

The Ga(P_1-x_,Bi_x_)/GaP sample studied in this work was grown by MOVPE using a commercially available Aixtron horizontal reactor system (AIX 200-reactor) with gas foil rotation. Triethylgallium (TEGa), tertiarybutylphosphine (TBP), and trimethylbismuth (TMBi) were used as precursors for Ga, P and Bi, respectively. The growth temperature for the Ga(P_1-x_,Bi_x_) layer was set to 400 °C, while the GaP barriers growth temperature was set to 625 °C to remove surplus Bi from the surface. Further information, including a more detailed description of the MOVPE growth procedure of the Ga(P_1-x_,Bi_x_)/GaP sample is summarized thoroughly by Nattermann *et al*.^14^. The sample used for the present study consists of two 55 nm thick Ga(P_1-x_,Bi_x_) layers with Bi fractions of x = 3.6% and x = 7.4%, separated by GaP barriers with an approximate thickness of 100 nm. The Bi fraction of each Ga(P_1-x_,Bi_x_) layer is determined using secondary ion mass spectrometry (SIMS), with complementary high resolution X-ray diffraction (HRXRD) measurements taken for comparative purposes. Here a HRXRD pattern was simulated around the (004)-GaP substrate peak, assuming the GaBi lattice constant to be 6.33 Å^3^. Due to the micrometer sized spot of the XRD, the resulting content values represent an average. Since potential clusters may exhibit a rhombohedral structure^16^ and their size is in the nanometer length scale, it is unlikely that they influence these (004) rocking curves at all. The sample preparation for the presented *in situ* study was done using a JEOL JIB 4601 F FIB system. The detailed preparation process of the lamella and the loading procedure of the finished specimen into the *in situ* cell will not be repeated here, but a detailed description can be found in Straubinger *et al*.^24^. In summary, an electron transparent lamella was prepared out of a wafer piece as described in Schaffer *et al*.^25^. Upon completion, the lamella was rotated by 90° along the long axis and mounted with the electron transparent section positioned precisely above the electron transparent SiN window of the thermal e-chip produced by Protochips. The STEM investigations were performed in a double-C_S_ corrected JEOL JEM 2200 FS field emission TEM operating at 200 kV. For the *in situ* investigation, a modified atmosphere system with a gas environmental cell holder from Protochips was used. A detailed description of the system, the specifications, and the required modifications are given in Straubinger *et al*.^26^. This setup allows for high resolution (S)TEM investigation of III/V semiconductor materials in the growth temperature regime and under the necessary group V stabilization, which is indispensable when aiming to avoid group V desorption. Image generation was carried out in high angle annular dark field (HAADF) mode, also known as Z-contrast mode^27,28^ due to the underlying Rutherford-like distribution of the scattered electrons. Hence, the detected intensity is proportional to Z^1.6–2^, which helps to intuitively distinguish between different elements. In order to allow for determining thickness in later stages, the acquired images were normalized with respect to the impinging beam following the procedure described in He & Li^29^. The HAADF intensity is corrected by subtracting the intensity contribution of the SiN windows from the measured intensity of the crystalline sample. This approximation is justified because both SiN windows are amorphous and highly defocused. Subsequent comparison with complementary contrast simulation using the frozen phonon approximation available in STEMsim^30^ was used to determine the lamella thickness. A more detailed description of all simulation parameters is given in^26^.

## Results and Discussion

This paper is organized as follows: first, by tracking the lamella thickness over the temperature for different TBP pressures in the *in situ* cell, it should be ensured that the following measurements are not influenced by any destructive behaviour of the crystal structure due to P desorption. Next, the initial Bi cluster formation temperature and the cluster characteristic in terms of cluster size and formation time is determined by analysing the intensity distribution within the Ga(P_1-x_,Bi_x_) layers for three temperature series covering the MOVPE growth temperature regime. Here, one question to be answered refers to the time during which the local Bi enrichment takes place in the growth process. Subsequent annealing studies at temperatures significantly above the growth temperature will ascertain the relationship between the Bi fraction in the Ga(P_1-x_,Bi_x_) layer and the initial Bi clustering temperature. Finally, a closer insight into the crystalline structure of the Bi clusters within the Ga(P_92.6_Bi_7.4_) layer will be given.

**Figure 1:**
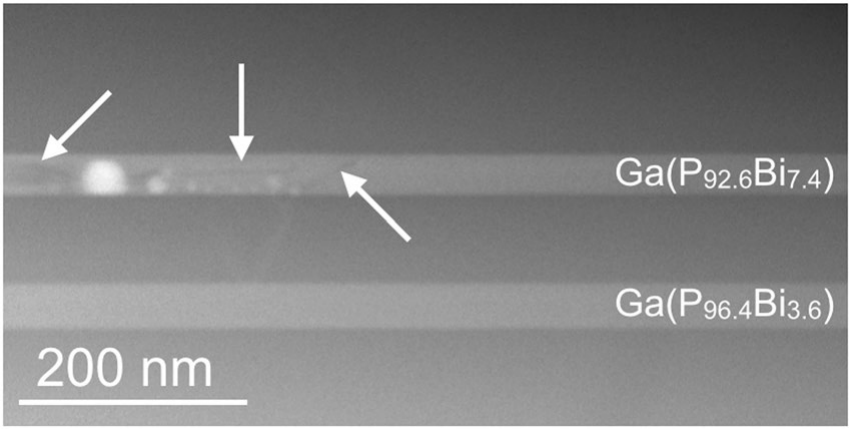
HAADF STEM overview image of the dedicated structure containing two 55 nm thick Ga(P_1-x_,Bi_x_) layers with different fractions of Bi embedded in approximately 100 nm thick GaP barriers. Some of the brighter appearing Bi clusters can be observed on the left hand side in the very top Ga(P_92.6_Bi_7.4_) layer. Around the brighter areas a contrast variation is observable which might originates from Bi depleted tracks left from moving Bi. (marked with white arrows).

Figure 1 shows a HAADF STEM overview image of the investigated structure as grown, i.e without thermal treatment. As mentioned in the experimental section the sample consists of two Ga(P_1-x_,Bi_x_) layers with a different Bi content. The Bi fraction is indicated on the right hand side of the image. Every Ga(P_1-x_,Bi_x_) layer has a thickness of around 55 nm and is surrounded by approximately 100 nm of GaP. Due to the large atomic number of Bi (Z = 83), the clusters appear brighter in the HAADF STEM image. Some Bi enriched areas can be observed on the left hand side in the very top Ga(P_92.6_Bi_7.4_) layer. Due to the thickness of the lamella and the resulting overlay of the rhombohedral crystal structure of a pure Bi cluster and the surrounding zincblende host material, it is not possible to determine whether the bright areas are rhombohedral Bi clusters or Bi enriched zincblende Ga(P_1-x_,Bi_x_). Therefore, we will refer to the bright areas within the Ga(P_1-x_,Bi_x_) layer as clusters in the following. This is justified because the post thermal annealing investigation at a thin sample region reveals that the bright areas, which occur due to the thermal annealing, consist of rhombohedral Bi^16^ as will be shown later. By taking a closer look at the areas around the brighter areas in Fig. 1 (some examples are marked by white arrows), a contrast variation is observable. These appearances might originate from Bi depleted tracks left from moving Bi during the cluster formation. Before investigation of the detailed characteristics of the Bi clusters, it should be clarified whether the cluster formation happens during the growth of the Ga(P_1-x_,Bi_x_) layer itself taking place at 400 °C or during the growth of overlying GaP layer acting as a 625 °C thermal annealing for the lower Ga(P_1-x_,Bi_x_) layers. To ensure no contribution of already existing Bi clusters, the experiments were carried out in areas with a homogeneous Bi distribution.

**Figure 2:**
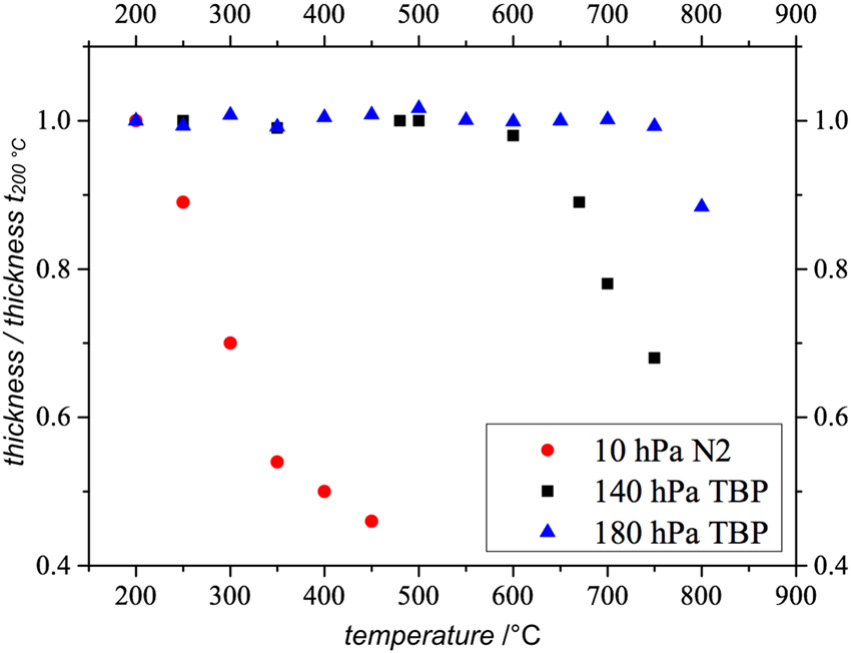
Plot showing the thickness divided by initial thickness (t_200 °C_) versus the temperature. The three measurements compare the non-destructive temperature regime of the GaP region of the TEM lamella without group V stabilization (red dots) with two different amounts of TBP (black squares 140 hPa TBP, blue triangles 180 hPa TBP) in the cell during the thermal annealing^26^.

To facilitate the investigation of III/V semiconductor materials *in situ* in the TEM at growth temperature regime, the crystal structure must be stabilized during the *in situ* annealing investigation to prevent group V desorption. This ensures that the observed changes do not occur due to structural degradation. One method to measure the sample destruction is to track the TEM lamella thickness during the *in situ* experiment. By doing so it is possible to identify the non-destructive temperature range in dependence of the group V stabilization. Figure 2 shows the results of these thickness measurements. Here, the thickness divided by the initial thickness (t_200 °C_) over the temperature is shown for an unstabilized environment and two different TBP pressures in the *in situ* cell. The red dots belong to the unstabilized annealing experiment, the black squares and blue triangles show the results of the thermal treatment experiments applying 140 hPa and 180 hPa TBP, respectively. It should be mentioned that the thickness measurement in the unstabilized experiment and in the annealing series with 140 hPa TBP in the cell was carried out on a different TEM lamella within a GaP region with approximately 3% B which is negligible in the analysis. By comparing the stabilized with the unstabilized experiment one can clearly see the tremendous difference in the sample stability during the thermal treatment. Furthermore, by comparing the thermal treatment experiment under 140 hPa TBP (black squares) and 180 hPa TBP (blue triangles) environments, the different non-destructive temperature regimes can be seen directly. With 180 hPa TBP in the cell the crystal structure is thermally stable up to 750 °C, whereas the sample annealed in the 140 hPa TBP environment starts to degrade at around 600 °C. As a result of these findings, all further *in situ* annealing investigations discussed in this paper are carried out under a 180 hPa TBP stabilization.

**Figure 3:**
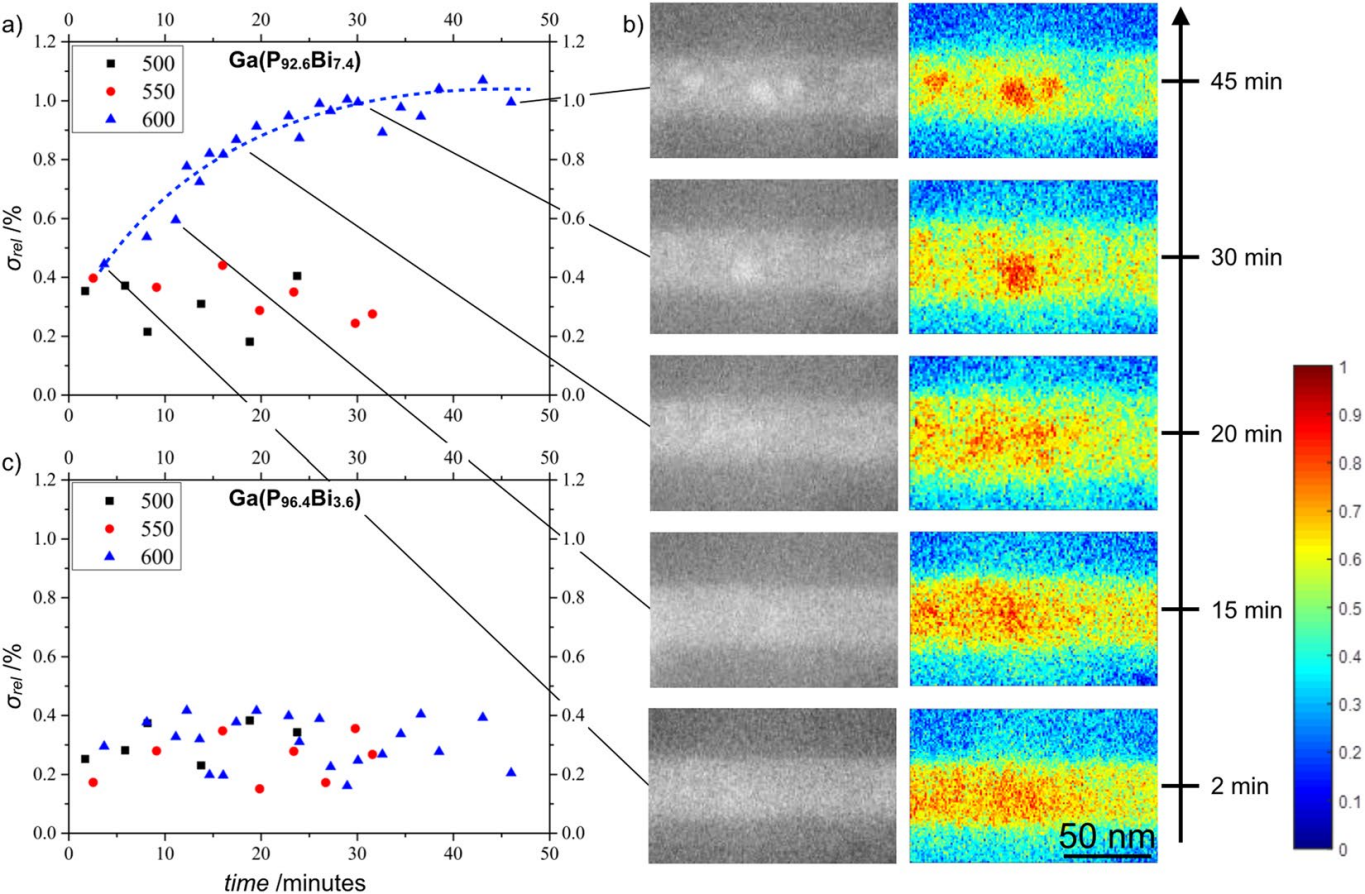
Plot (**a**) and (**c**) show σ_rel_, i.e. standard deviation of the intensity within the ternary Ga(P_1-x_,Bi_x_) layer divided by the mean intensity of the surrounding GaP matrix, over the time within the Ga(P_1-x_,Bi_x_) layer with 7.4% and 3.6% Bi, respectively. The temperature series were carried out at 500 °C (black squares), 550 °C (red dots) and 600 °C (blue triangles). The original as well as the color coded STEM images (**b**) show the formation of one big and two smaller Bi clusters within the Ga(P_92.6_Bi_7.4_) layer over time. The color-coding scale is shown on the far right hand side.

**Figure 4:**
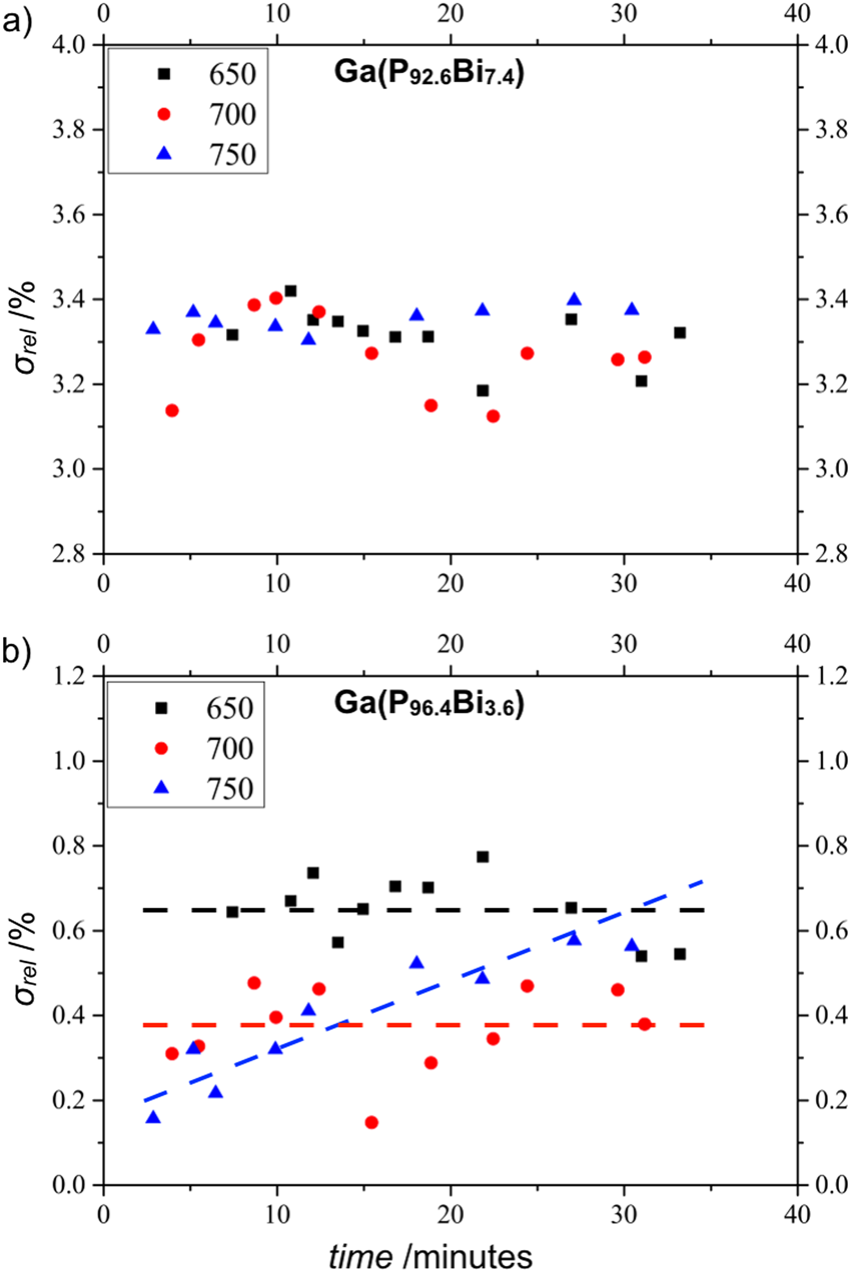
Plot (**a**) and (**b**) show σ_rel_ over the time within the Ga(P_1-x_,Bi_x_) layers with 7.4% and 3.6% Bi. These measurements were performed at 650 °C (black squares), 700 °C (red dots) and 750 °C (blue triangles).

**Figure 5:**
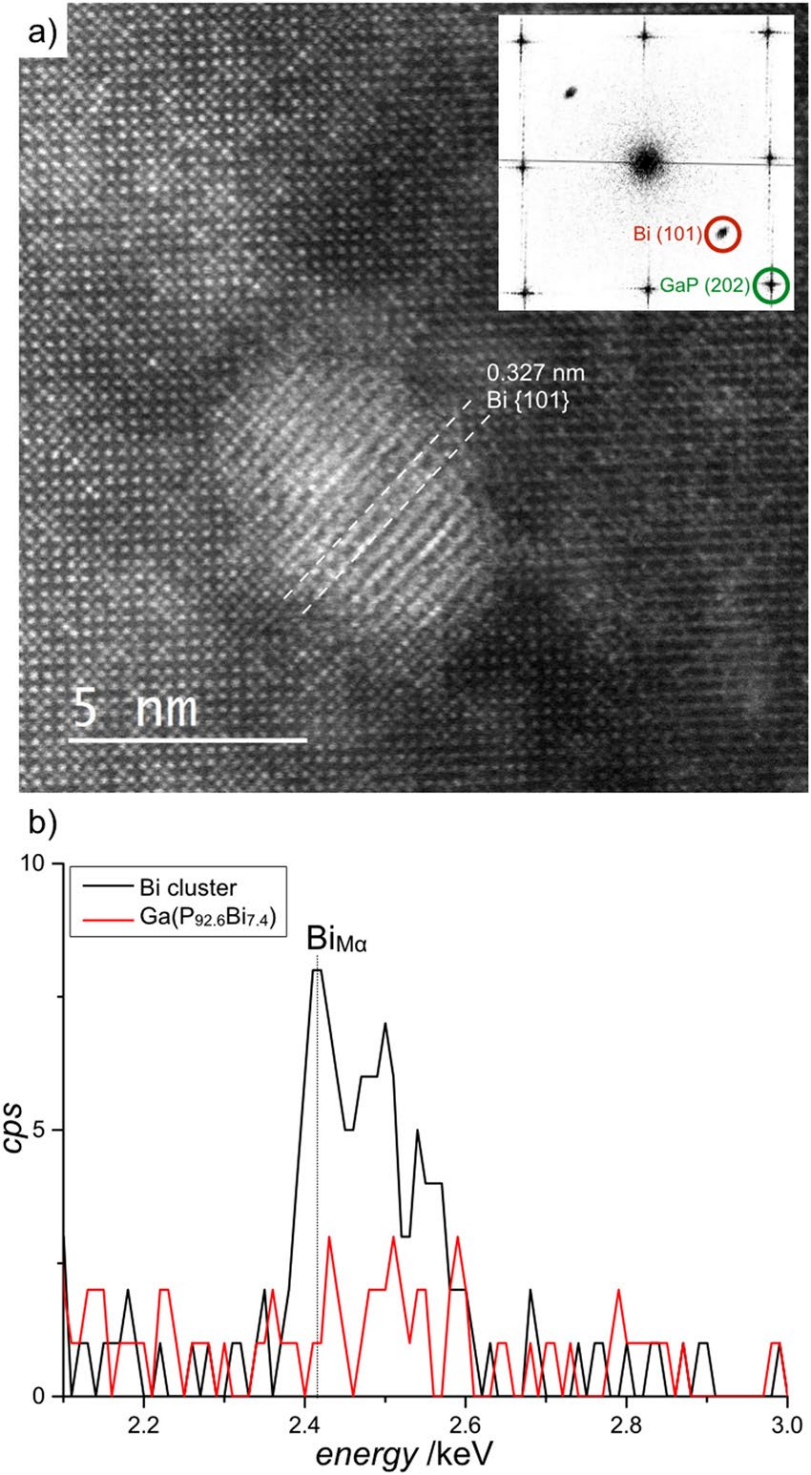
Atomic resolution HAADF STEM image of the Ga(P_92.6_Bi_7.4_) layer. The brighter region in the middle of the image shows a Bi cluster embedded in the Ga(P_92.6_Bi_7.4_) layer. The inset shows the inverted FFT of the STEM image shown in (**a**). Plot (**b**) shows the EDS result of the Bi_Mα_ edge derived from the region at the Bi cluster (black line) and from a region without any local Bi enrichment (red line).

To identify the initial temperature at which the clustering takes place the temperature was gradual increased by 50 °C starting with 300 °C which is significantly below the growth temperature of 400 °C and 625 °C, respectively. For every series the temperature was kept for 30 to 45 minutes. The HAADF STEM images recorded during every series in an approximately two minute cycles are the basis for the Bi clustering study within the Ga(P_1-x_,Bi_x_) layer. The measured standard deviation of the intensity within the ternary Ga(P_1-x_,Bi_x_) layer divided by the mean intensity of the surrounding GaP matrix, hereinafter designated as σ_rel_, is a measure for the inhomogeneity of the Bi distribution of the corresponding Ga(P_1-x_,Bi_x_) layer, as has been shown on the example of Ga(N,As,P) by Wegele and co workers^31^. It should be pointed out that due to the large difference in the surface to bulk ratio and the relaxed strain in the thin lamella, the absolute value for the initial temperature above which the clustering takes place in the MOVPE process and inside an electron transparent TEM lamella are not directly comparable. Nevertheless, the trend is the same and therefore the results presented in the following allow an *in situ* investigation of a specific MOVPE process inside the STEM under high resolution conditions. Plot (a) in Fig. 3 shows the σ_rel_ within the Ga(P_92.6_Bi_7.4_) layer gained from the aforementioned HAADF STEM images over the time for three temperature steps. By taking a look at the data points of the 500 °C time series (black squares), no increase in σ_rel_ can be observed. This indicates that no local Bi enrichment takes place. It should be mentioned that this temperature is already 100 °C above the Ga(P_1-x_,Bi_x_) layer growth temperature of 400 °C. Also, by further increasing the temperature by 50 °C to 550 °C (red dots) no change in the structure is visible. The blue triangles represent the time series at 600 °C, here the influence of the temperature on σ_rel_ within the Ga(P_92.6_Bi_7.4_) layer which originates from Bi clustering can be seen. It is worth noting that due to the random Bi distribution within the Ga(P_1-x_,Bi_x_) layer, the fluctuation of the sample thickness, and the presence of amorphous layers during sample preparation, the value of σ_rel_ is not zero even at the beginning of the experiment. It should also be emphasised that post *in situ* energy dispersive X-ray spectroscopy (EDS) measurements shown in Fig. 5(b) prove that the observable bright areas consists of Bi and do not consist of Ga which might form Ga droplets on the surface as observed in destructive temperature regimes. To better illustrate the process of Bi clustering a dashed line as a guide to the eye is added to the annealing series done at 600 °C in Fig. 3(a). Taking a closer look at the curvature of this line, the time course of the structural change is directly observable. The clustering of Bi within the Ga(P_92.6_Bi_7.4_) layer takes around 40 minutes before the clusters reach a stable size. Later investigation, shown in Fig. 4, at higher temperatures will clarify whether the cluster size increases further with rising temperatures or if there is a stable Bi cluster formation over the whole temperature regime above the threshold temperature. The STEM images presented in Fig. 3(b) show the formation of one big and two smaller Bi clusters during the temperature series at 600 °C. It is worth noting that the very long investigation time of this experiment and the presence of the group V precursor gas in the *in situ* cell resulted in a carbon coating forming over time, which caused a reduction of the contrast in the STEM images. Therefore, the HAADF intensity in the images presented on the left hand side of Fig. 3(b) are the original STEM images and are presented with the same intensity scale. For comparison, the micrographs shown on the right hand side of Fig. 3(b) are color-coded from blue (min. Intensity) to red (max. intensity) and in their individual intensity scale, since it enhances the visibility of the clusters. The scale of color-coding is shown on the far right hand side. The time passed with respect to the start of the experiment at which each image is recorded is indicated at the very right hand side. Furthermore, the black lines between the plot (a) and (b) relate the STEM image with the corresponding data point. It should be mentioned that the STEM images in Fig. 3(b) show only a part of the larger images investigated to generate the data points presented in Fig. 3(a). To further compare the Bi clustering in dependence of the Bi fraction, plot (c) in Fig. 3 shows σ_rel_ drawn over the time within the Ga(P_96.4_Bi_3.6_) layer. Here, no Bi clustering could be observed for all three temperature series, i.e. 500 °C, 550 °C, and 600 °C. Overall, the results presented in Fig. 3 already answer the question regarding the time during which the local Bi enrichment occurs within the growth process. It thus can be concluded that the Ga(P_92.6_Bi_7.4_) layer structure might grow without Bi clusters, and due to the subsequent GaP layer which has a growth temperature of 625 °C, the inhomogeneity of the Bi distribution takes place. This result is further supported by *ex situ* investigations on Ga(P_92.6_Bi_7.4_) layers without a GaP cap, where no Bi clusters appear in the structure. Further experiments significantly above the growth temperature should demonstrate the relationship between the temperature at which Bi clustering takes place and the Bi fraction in the Ga(P_1-x_,Bi_x_) layer as well as if the cluster size in the Ga(P_92.6_Bi_7.4_) layer further increases with rising temperature.

To further investigate the initialisation temperature with regard to the Bi fraction, the plots in Fig. 4 show σ_rel_ for the same Ga(P_1-x_,Bi_x_) layers presented in Fig. 3 but at temperatures significantly above the growth temperatures. It should be noted that the measurements of the Ga(P_92.6_Bi_7.4_) layer were carried out at a thinner section on the lamella, therefore the values of σ_rel_ are slightly different compared to the values in Fig. 3(a). However, a closer look at the plot (a) in Fig. 4 reveals that there is no further inhomogeneity in the Bi distribution caused by increasing the temperature from 650 °C up to 750 °C. This confirms the previous observation that the Bi cluster formation reaches a stable size which is steady against higher temperatures. Due to the large spreading of the data points presented in Fig. 4(b), dashed lines are added to the Ga(P_96.4_Bi_3.6_) layer measurements as a guide to the experiment. By comparing the series recorded at 650 °C (black squares) and the series measured at 700 °C (red dots), one cannot see any hint of Bi clustering which would lead to an increase of σ_rel_. In contrast to that, it can be assumed that there is a rising σ_rel_ value in the annealing series recorded at 750 °C within the Ga(P_96.4_Bi_3.6_) layer. From that point of view one can speculate that the Bi fraction in the Ga(P_1-x_,Bi_x_) layers is directly related to the initial clustering temperature. Unfortunately, it is not possible to further increase the temperature to investigate the Bi clustering in the Ga(P_96.4_Bi_3.6_) layer without destroying the crystal structure in a 180 hPa TBP environment. Further studies carried out at higher TBP pressures will investigate this correlation in more detail.

Figure 5(a) shows a atomic resolution HAADF STEM image of one Bi cluster recorded in the Ga(P_92.6_Bi_7.4_) layer after the thermal annealing experiment. To have tilting capability in both directions and orientate the sample precisely along the [010] viewing direction, the image is recorded in a standard double tilt holder. Here, the lattice planes corresponding to rhombohedral single crystal Bi are clearly resolved. The single crystalline lattice parameter for Bi were determined and published by D. Schiferl and C.S. Barrett^32^. Two rhombohedral Bi {101} planes with a spacing of 0.327 nm are highlighted in Fig. 5(a) with white dashed lines. The inset in Fig. 5(b) shows the inverted FFT of the STEM image shown in Fig. 5(a). The FFT was calibrated using the {200} reflections of Ga(P,Bi) which retain the spacing of pure GaP due to the tetragonal distortion. Here the parallelism between the Bi {101} and the Ga(P,Bi) {202} planes is clearly visible. The aforementioned contrast variation resulting from Bi depleted regions due to migrating Bi during the formation of Bi cluster can also seen in the STEM image in Fig. 5(a) at the lower right edge of the Bi cluster. The preferred moving direction seems to be along the [−101] and [101] direction. This was also observed in STEM images at higher magnifications. The plot in Fig. 5(b) shows the EDS results of the region at the Bi cluster (black line) and for comparison at a region within the Ga(P_92.6_Bi_7.4_) layer but without Bi clusters (red line). The counts per second (cps) over the energy in keV is plotted in the energy range around the Bi_**Mα**_ edge. This result proves that the bright areas in the STEM images consist of Bi.

## Summary

In this paper, we first demonstrate that the used *in situ* setup facilitates the investigation of III/V semiconductor materials in a TEM in the growth temperature regime since it provides the necessary group V stabilization. Moreover, we determine the temperature above which the Bi clustering takes place. Based on the two investigated composition values, we find that this initial temperature depends on the actual Bi fraction within the Ga(P_1-x_,Bi_x_) layers. By investigating the Ga(P_1-x_,Bi_x_) layers at even higher temperatures, we were able to conclude that there is no further Bi rearrangement. The process is most likely limited by the Bi amount available in the thin TEM lamella. Comparing these results to the findings of the as grown sample suggests that the Bi clustering during the MOVPE growth process most likely took place during the growth of the subsequent GaP layer which acts as a 625 °C thermal treatment of the lower Ga(P_1-x_,Bi_x_) layer. Finally, a closer look at a representative Bi cluster reveals its rhombohedral structure and its orientation relation to the Ga(P,Bi) matrix.
